# A Novel Effect of Microaneurysms and Retinal Cysts on Capillary Perfusion in Diabetic Macular Edema: A Multimodal Imaging Study

**DOI:** 10.3390/jcm14092985

**Published:** 2025-04-25

**Authors:** Bilal Haj Najeeb, Bianca S. Gerendas, Alessio Montuoro, Christian Simader, Gábor G. Deák, Ursula M. Schmidt-Erfurth

**Affiliations:** Vienna Reading Center, Department of Ophthalmology, Medical University of Vienna, Waehringer Guertel 18-20, 1090 Vienna, Austria

**Keywords:** diabetic macular edema, diabetic retinopathy, fluorescein angiography, optical coherence tomography, retinal non-perfusion, microaneurysms

## Abstract

**Background/Objectives:** The aim of this study was to investigate the potential contribution of microaneurysms (MAs) and retinal cysts to the pathogenesis of macular non-perfusion in patients with diabetic macular edema (DME) using multimodal imaging. **Methods:** In this cross-sectional study, 42 eyes with DME were analyzed using color fundus photography, fluorescein angiography (FA) and optical coherence tomography (OCT). Macular non-perfusion within the central 3000 µm was categorized by location and extent into foveal avascular zone enlargement (FAZE), focal non-perfusion (FNP) and diffuse non-perfusion (DNP). A custom-developed software was used to assess the colocalization of retinal cysts on OCT with areas of non-perfusion on the corresponding FA images. Also, the presence of leaky MAs adjacent to retinal cysts on FA was verified. **Results:** Colocalization between retinal cysts and non-perfusion was observed in 32 of 42 (76%) eyes: 19 of 23 (83%) eyes with FAZE and 13 of 16 (81%) eyes with FAZE+FNP. No cysts colocalization was found in all three eyes (100%) presenting with DNP. None of the eyes presented with FNP alone. In the remaining seven eyes (four eyes with FAZE and three eyes with FAZE+FNP), no colocalization was noticed. At least one leaky MA adjacent to retinal cysts was identified in all eyes presented with colocalization. **Conclusions:** Retinal cysts may contribute to the development of limited non-perfusion in DME. Leaky MAs appear to be the primary source of cyst formation, which may lead to localized capillary occlusion in the macula.

## 1. Introduction

Capillary non-perfusion is one of the main manifestations of diabetic retinopathy (DR) [1]. It represents the transitional step of non-proliferative occlusive and inflammatory vascular changes to the formation of the most ominous complications, such as retinal neovascularization and vitreous hemorrhage. One in five patients with diabetes mellitus without clinical retinopathy presents with capillary non-perfusion [2]. Diabetic macular edema (DME), which is manifested as fluid extravasation due to the disruption of the inner blood retinal barrier (iBRB), is another leading cause of visual loss in patients with DR and often occurs alongside the development of non-perfusion [3,4].

Non-perfusion and DME were recently investigated using optical coherence tomography (OCTA), revealing a colocalization between central cysts and non-perfusion areas [5]. However, the study did not explore any interaction between these two pathologies, nor did it evaluate the potential contribution of adjacent microaneurysms (MAs). Subsequently, in the present work, we apply a multimodal imaging approach to investigate whether central cysts could contribute to capillary non-perfusion by occluding the surrounding capillary network in the inner nuclear layer (INL). Furthermore, we explore the potential role of leakage from adjacent MAs in the pathogenesis of non-prefusion.

The background of our study is based on histological and imaging studies indicating that the INL contains the centrally located, solitary capillary layer that delineates the foveal avascular zone (FAZ). Beyond the FAZ boundary, the INL comprises a double lamina of the capillary network (intermediate and deep capillary plexuses) [6,7,8].

## 2. Methods

### 2.1. Patients and Data Collection

The data were derived from prospective, randomized, double-blinded, multi-center international clinical trials that evaluated different forms of anti-vascular endothelial growth factor (VEGF) treatment in patients with type 1 or 2 diabetes mellitus and DME. Informed consent was obtained from all participants of the original trials. In this post hoc analysis, we selected 42 consecutive eyes at baseline from the imaging database of the Vienna Reading Center (VRC). This study was performed in adherence to the tenets of the Declaration of Helsinki, and approval for post hoc data analysis was obtained from the institutional review board of each participating center and of the Medical University of Vienna. The independent and standardized image analysis of these studies was carried out by two certified image graders at the VRC. In case of disagreement, the decision was taken by a retina specialist (BHN). All patients underwent baseline imaging that included OCT, color fundus photography and FA with a minimum resolution of 1024 × 1024 pixels. Only baseline images of the study eyes were enrolled in this analysis.

### 2.2. Inclusion and Exclusion Criteria

Inclusion criteria: Presence of DME with a central subfield thickness of more than 300 µm on OCT, as defined by the study protocols from which the cohort was derived. Also, evidence of macular non-perfusion on FA involving at least the central 3000 µm (the central and inner subfields of the ETDRS grid).

Exclusion criteria: Treatment with vascular endothelial growth factor inhibitors within 90 days or corticosteroid within 120 days prior to imaging, presence of active proliferative DR, patients with uncontrolled hypertension, uncontrolled diabetes (HbA1c > 12%), coexisting retinal diseases that may interfere with the diagnosis or analysis such as retinal vascular occlusion, presence of other clinical findings that could obscure morphological changes on OCT or FA, including extensive lipid deposition (dense exudate), hemorrhage, pigment clumping or scarring.

### 2.3. Registration of OCT and Early-Phase FA Images

Using a custom-developed software, we registered each set of horizontal OCT B-scans with their corresponding early-phase FA image. Our self-developed image registration software was used in an interactive process, in which FA images were overlaid with enface OCT images. The registration was not fully automated but was visually verified by experienced graders. A successful registration was defined by the alignment of at least three common anatomical landmarks such as vessel bifurcations, dot hemorrhages and microaneurysms across the two modalities, followed by automatic superimposition of the FA image over the enface image. Thus, the chain of consecutive OCT B-scans of a specific area of the macula could be visualized and assessed by scrolling on the edited FA image, which, in turn, showed the area of non-perfusion for the same macular area. This approach was applied in all cases to ensure that the analysis was based on reliably co-registered image data. Editing tools were utilized to enhance the assessment of the images. The foveal center point and the central 3000 µm area were determined on the OCT scans by certified OCT graders from the VRC, who were masked to the corresponding FA images.

### 2.4. Types of Non-Perfusion

The presence and extent of non-perfusion were determined based on the standard images of non-perfusion on FA in the ETDRS study [9]. Non-perfusion within the central 3000 µm has been classified into three distinct types according to its location and extent on FA.

**Figure 1:**
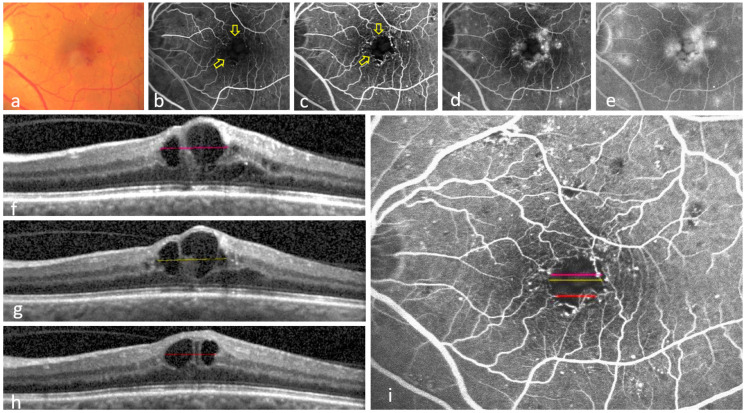
Foveal avascular zone enlargement (FAZE) in diabetic macular edema (DME). A color fundus photograph shows multiple red intraretinal hemorrhages and yellow hard exudates in the left eye of a patient with DME (**a**). Two fluorescein angiography (FA) images from the first minute of the angiogram demonstrate the FAZE as an enlarged central hypofluorescent area with a horizontal diameter of 750 µm, and microaneurysms (MAs) as focal hyperfluorescence (**b**,**c**). Note the apparent accumulation of MAs around the outline of the FAZE, except in the superior and inferior nasal areas (yellow arrows) (**c**). A 2 min FA image shows that leakage, visible as hyperfluorescence of the cystoid spaces, predominantly originates from the MA-rich perifoveal area (**d**). A 10 min FA image depicts cystoid DME (petaloid pattern of hyperfluorescence) (**e**). Three colored line measurements on three optical coherence tomography (OCT) B-scans indicate the dimensions of the cysts occupying the inner nuclear layer (**f**–**h**). These line measurements are simultaneously displayed on the corresponding early FA image (**c**) after registration (**i**). Note that the extent of these cysts in the OCT B-scans (**f**–**h**) closely corresponds with the area of FAZE in the early FA images in (**i**), suggesting a causative relationship. Also note the absence of increased vascular density around the FAZE in (**i**). The scarce fluorescence of fine arterioles at the temporal end of the three colored lines in (**i**) is a result of patent end vessels in the overlying retinal layers, as the outer retina is devoid of capillaries.

1: Foveal avascular zone enlargement (FAZE): An increased horizontal FAZ diameter (>650 µm) in early-phase FA images due to the occlusion of the centrally located solitary capillary layer that delineates the FAZ within the INL (Figure 1) [10].

**Figure 2:**
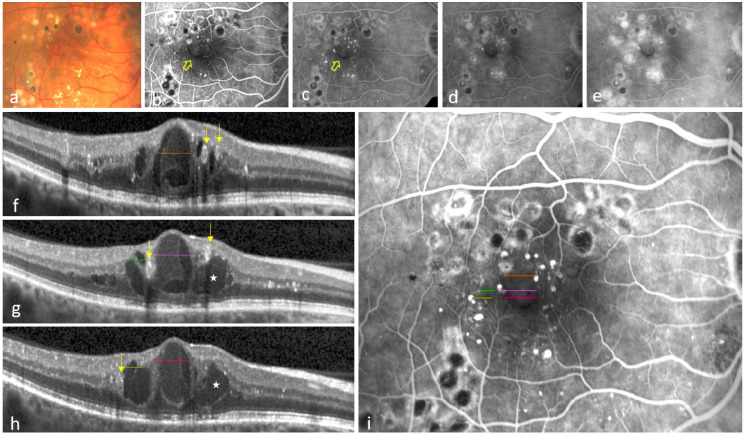
Foveal avascular zone enlargement (FAZE) and focal non-perfusion (FNP) in diabetic macular edema (DME). A color fundus photograph illustrates intraretinal hemorrhages, hard exudates and previous laser treatment in the right eye of a patient with DME (**a**). Two consecutive fluorescein angiography (FA) images from the first minute of the angiogram show microaneurysms (MAs) as focal hyperfluorescent spots around FNP and FAZE, except in the inferior region (yellow arrow) (**b**,**c**). A 5 min FA image reveals leakage, which primarily emerges from the MA-rich capillaries (**d**). A late 10 min FA image depicts a petaloid pattern of cystoid DME (**e**). Three optical coherence tomography (OCT) B-scans (**f**–**h**) through the central macula demonstrate cysts and their correlation with the extent of non-perfusion on the corresponding FA image (**b**) after registration (**i**). The brown, violet and red lines through the central cyst in (**f**–**h**) correspond to the extent of the area of FAZE in (**i**). Similarly, the green and yellow lines in (**g**,**h**) closely correspond with the area of FNP in (**i**), where the inner nuclear layer (INL) is completely occupied by the central cysts. Note that the large cyst marked with an asterisk (_*_) in (**g**,**h**) was excluded from the evaluation, as it does not fully occupy the INL. Also note the proximity of the underlying MAs (arrows) identified in (**f**–**h**), which suggests a causal relationship. Moreover, the absence of increased vascular density around the areas of non-perfusion (FAZE, FNP) rules out the lateral displacement of capillaries as a mechanism of non-perfusion.

2: Focal non-perfusion (FNP): A perifoveal, circumscribed abnormal increase in intercapillary distance, attributed to damage of the intermediate and deep capillary plexuses, which are located on either side of the INL (Figure 2).

**Figure 3:**
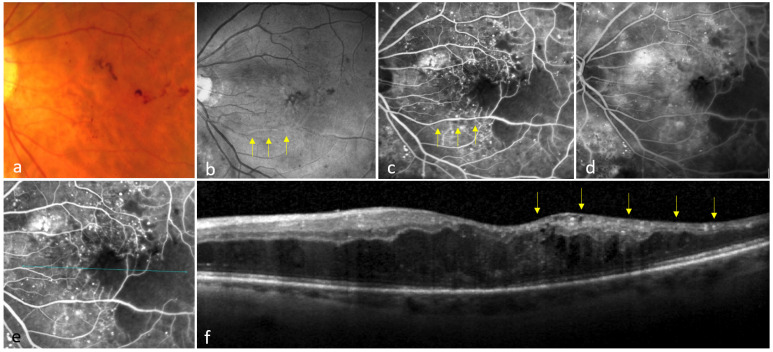
Diffuse non-perfusion in diabetic macular edema (DME). A color fundus photo shows intraretinal hemorrhages in the left eye of a patient with DME (**a**). The red-free image shows obscuration of the inferior temporal retinal artery (arrows) (**b**). An early-phase fluorescein angiography (FA) image reveals widespread ischemia involving both the central and outer macula (**c**). Note the irregular diameter of the inferior temporal artery (arrows), attributed to intraluminal atherosclerosis (**c**). A 10 min FA image demonstrates leakage and macular edema (**d**). An optical coherence tomography (OCT) scan (blue line) is shown on FA image (**c**) after registration (**e**). The OCT B-scan presents generalized thinning of the ischemic inner retina (arrows) with no inner nuclear layer cysts that could be matched with the non-perfusion area on FA (**f**).

3: Diffuse non-perfusion (DNP): A widespread area of capillary non-perfusion, characterized by ischemia extending beyond the central 3000 µm (Figure 3).

### 2.5. MA

Recognition of MAs was based on FA, where they appeared as sharply demarcated hyperfluorescent focal lesions along the retinal vessels. A leaky MA was determined by a steady increase in hyperfluorescence over time, originating from the MA itself.

Contrary to FA performed in clinical practice, the acquisition protocol of the included studies captured at least 10 images of the study eye during the first minute of the angiogram (early-phase images). This cascade of images enabled the graders to determine whether the hyperfluorescence observed within retinal cysts originated from neighboring MAs (Figure 1 and Figure 2).

### 2.6. Morphologic Image Analysis

A colocalization was considered to be present under the following conditions: (1) in eyes with FAZE, when the lateral extension of foveal cysts in the outer retina and the perifoveal cysts in the INL on OCT are located within the boundary of the FAZE in early-phase FA; (2) in eyes with FNP or DNP, when the cysts extended vertically to involve at least the INL, with the preservation of the overlying inner retinal layers on OCT, and remained confined within the area of non-perfusion on FA. Furthermore, in eyes demonstrating colocalization, we investigated whether at least one leaky MA was present in early-phase images, resulting in hyperfluorescence of the neighboring cyst in late-phase images of FA.

## 3. Results

**Figure 4:**
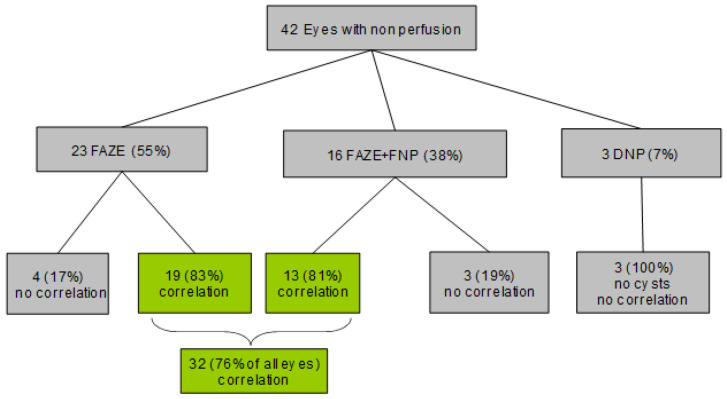
Flow diagram showing the characteristics of non-perfusion types in 42 eyes based on their correspondence with cysts in the retinal layers. FAZE: foveal avascular zone enlargement; FNP: focal non-perfusion; DNP: diffuse non-perfusion.

We included 42 consecutive eyes from 42 patients, selected according to our inclusion/exclusion criteria. The three types of non-perfusion, classified based on the vertical location of the cysts and the horizontal extent of the non-perfusion areas, were identified as follows: 23 (55%) eyes exhibited FAZE, 16 (38%) eyes showed a combination of FAZE and FNP (FAZE+FNP), and 3 (7%) eyes demonstrated DNP. No eyes with FNP only were found (Figure 4).

### 3.1. Eyes Showing at Least One Colocalization of INL-Occupying Cysts and Non-Perfusion Areas

FAZE group: A total of 19/23 eyes (83%) showed colocalization between the existence of cysts on OCT and the outline of FAZE on FA. These cysts extended from the outer plexiform layer/outer nuclear layer (OPL/ONL) into the inner retina centrally and occupied at least the full thickness of the INL on OCT (Figure 1).

FAZE+FNP group: A total of 13/16 (81%) showed, foveally, the same colocalization seen in the FAZE only group. In addition, perifoveally, there were cysts that had a breakthrough into at least the INL, with the preservation of the overlying inner retinal layers on OCT (Figure 2).

The total number of eyes showing at least one colocalization of INL-occupying cysts and non-perfusion areas was 32/42 (76%) of all eyes.

### 3.2. Eyes Showing No Colocalization Between INL-Occupying Cysts and Non-Perfusion Areas

DNP group: All 3/3 (100%) eyes exhibited generalized thinning associated with the loss of differentiation of the inner retinal layers. In addition, there was swelling of the outer retina. No colocalization between the location of cysts in the INL on OCT and the non-perfusion on FA was observed (Figure 3).

Remaining eyes: In the residual seven eyes (four eyes with FAZE, three eyes with FAZE+FNP), no colocalization was found. In these cases, the spatial extent of cysts on OCT did not respect the outline of non-perfusion areas on FA.

### 3.3. Distribution of MA

In all 32/32 (100%) eyes with at least one colocalization of both pathologies, there were one or more leaky MAs close to the cysts and non-perfusion areas. These MAs were obvious in early-phase FA images and leaked steadily over time, eventually contributing to cysts (Figure 1b–e and Figure 2b–e). Some MAs were also detectable on corresponding OCT B-scans (Figure 2f–h).

## 4. Discussion

In this study, we report on the potential influence of cysts, as imaged by OCT, on the development of capillary non-perfusion, as proved by FA. We found that both pathologies are colocalized in at least one area in over 75% of eyes with FAZE, with or without FNP.

**Figure 5:**
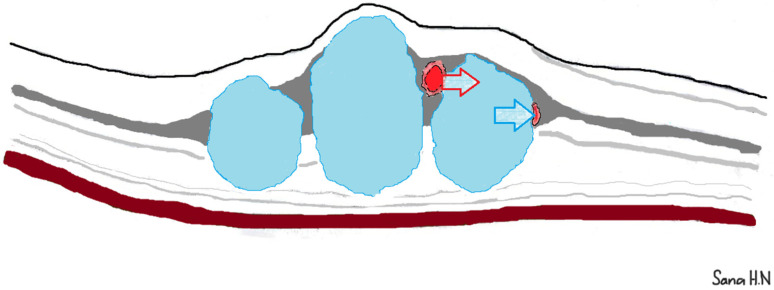
Proposed mechanism of non-perfusion in diabetic macular edema. The red arrow indicates the direction of fluids emerging from a microaneurysm located in the swollen inner nuclear layer. The high and sustained hydrodynamic pressure within the cysts (blue arrow) is hypothesized to induce the occlusion of the lumen of an adjacent capillary.

This characteristic coexistence could be explained by previous histological and OCT findings as the INL, where leaky MAs primarily emerge, is surrounded by retinal layers, which serve as high-resistance barriers to fluid extension [11]. As a result, retained edema from leaky MAs tends to expand locally, forming cysts. The increased hydrodynamic pressure within these cysts can occlude the adjacent capillary network within the INL. Subsequently, this damage will be manifested as limited non-perfusion on FA (Figure 5) [12].

However, we think that this extravascular effect has only a minor role in the development of non-perfusion in DME when compared to intravascular factors, such as atherosclerosis and diabetic microangiopathy.

This proposed mechanism supports our observation, made prior to the advent of OCTA, suggesting that a steady increase in cyst volume may cause an ab externo mechanical closure of retinal capillaries of 4–7 µm diameter [13]. It is also in agreement with a recent study demonstrating lateral displacement of capillaries due to the expansion of retinal fluid in exudative ME [12]. Moreover, in DME, damage to non-vascular (neural) retinal structures has been suggested as a result of the same hydrodynamic mechanism [14,15]. This mechanism may also explain the formation of macular hole in severe DME and why the macular non-perfusion is more extensive in DME patients compared to those with DR without DME [16,17].

Other retinal vascular diseases demonstrated a similar effect of the cysts. Hayreh showed angiographically how a massive macular edema can cause a selective central macular non-perfusion following central retinal artery occlusion [18,19]. This special form of ischemia, named “no-reflow” phenomenon, was consistently accompanied by significantly increased retinal thickness and photoreceptor disruption [19]. Obviously, the damage to the underlying photoreceptors is not due to deprivation of blood supply per se, as they are supplied by diffusion from the choriocapillaris, but rather appears to be a consequence of the massive retinal edema. Likewise, in retinal vein occlusion, which is also an acute event, non-perfusion was identified at the cystoid ME region, which persists after the resolution of the retinal swelling under anti-VEGF treatment [20]. In addition, our recent observation has yielded a comparable capillary damage in a certain type of neovascular age-related macular degeneration, which typically presents with severe cystoid ME [21,22].

MAs, defined as a focal breakdown of the iBRB, represent the earliest clinical feature of DR [23,24,25]. They are the main source of cystoid DME and preferentially emerge in the macular INL, often in association with FAZE (Figure 1 and Figure 2) [4,26,27,28]. Therefore, the presence of cysts originating from leaky MAs, with dimensions similar to the non-perfusion areas, very likely refers to a certain sequence of events: Leaky MAs induce the growth of cysts that occlude the adjacent capillaries in the INL, resulting in restricted non-perfusion (FAZE/FNP). This proposed mechanism is further supported by clinical evidence showing functional and morphological improvement in DME under laser photocoagulation of selected leaky MAs [29].

A previous OCTA study also illustrated a similar colocalization of non-perfusion areas and retinal cysts [5]. Nevertheless, OCTA does not capture the dynamic changes in fluid over time, nor does it clearly identify MAs as FA did in our study. These dynamic changes were essential to unravel the plausible sequence of pathophysiological events.

An opposing hypothesis has suggested that non-perfusion may predispose to cyst formation [30]. However, this theory fails to explain the development of edema within the FAZ, which is not affected by retinal perfusion impairment, as it derives its supply from the underlying choroid. Additionally, pericyte loss, a prerequisite for microaneurysm development, precedes the development of major intravascular occlusive factors, such as platelet aggregation and endothelial cell dysfunction [31].

Although FAZE and FNP may not involve extensive regions in the macula, their sequelae can be severe and irreversible. For instance, a deprivation of blood supply to the laterally displaced bipolar cells in the INL leads to an interruption in transmission of the high-resolution central vision generated by the foveal cones [14]. This effect may explain the worse visual function when the cysts are located in the INL compared to those situated in vessel-free layers such as the OPL [32]. To note, the reperfusion of occluded capillaries following treatment is still a subject of controversy [5,12,33].

Eyes with DNP showed no colocalization of both pathologies, but rather a generalized thinning and absence of cysts, coupled with a loss of differentiation in the inner retina. This finding is consistent with the results of a previous work, which suggests that intravascular occlusive factors, such as endothelial cell dysfunction and platelet aggregation, are the main cause of non-perfusion in this form [34,35]. The pathogenesis of capillary occlusion in DNP is intraluminal and involves all laminated retinal capillaries (Figure 3).

The non-perfusion observed in the remaining seven cases and other retinal areas of eyes with FAZE, with or without FNP, where neither colocalization nor cysts were present, could represent either a mixed form of intra- and extravascular mechanisms or an unstable volume of cysts resulting from previous anti-VEGF or corticosteroid treatment.

The absence of OCTA could be a limitation of our work. However, OCTA was not carried out in clinical trials from which our cohort was derived, and it is inferior to FA in illustrating the source and extension of leakage. Also, it is inconclusive in delineating MAs and capillaries with slow blood flow [36]. Moreover, it produces false decorrelation signals and projection artifacts that may interfere with a correct estimation of vascular density [36,37]. Furthermore, most OCTA machines falsely segment the vascular plexuses, in particular when severe edema is present, as in our cohort [38]. Recruiting participants from clinical trials could introduce selection bias. Another limitation is the absence of certain demographic and clinical characteristics of our patients. This is due to the fact that, in accordance with the study protocols of the prospective randomized clinical trials used in our analysis, graders at the VRC were masked to these details. The inclusion of eyes with longstanding DME is also a limitation. Additionally, the small sample size in certain groups and the cross-sectional design of this study limit the ability to establish causality. Therefore, a longitudinal study is needed to confirm a causative relationship. Nevertheless, the availability of 10 images from the first minute of the angiogram, along with the precise registration of FA and OCT images, allowed us to suggest the aforementioned sequence of pathological events.

## 5. Conclusions

Using a multimodal imaging approach, we report that MAs may play a key role in the development of limited non-perfusion in the central macula by inducing the formation of cysts within the INL.

## Data Availability

The data that support the findings of this study are not publicly available due to privacy reasons but are available from the corresponding author upon reasonable request.
